# RNA-Seq analysis of soft rush (*Juncus effusus*): transcriptome sequencing, de novo assembly, annotation, and polymorphism identification

**DOI:** 10.1186/s12864-019-5886-8

**Published:** 2019-06-13

**Authors:** Muhammad Arslan, Upendra Kumar Devisetty, Martin Porsch, Ivo Große, Jochen A. Müller, Stefan G. Michalski

**Affiliations:** 1 Department Environmental Biotechnology, Helmholtz Centre for Environmental Research – UFZ, Permoserstr, 15 Leipzig, Germany; 2 Institute for Biology V (Environmental Research), RWTH Aachen University, Templergraben 55, 52062 Aachen, Germany; 3 BIO5 Institute, The University of Arizona, Tucson, AZ 85719 USA; 4 Institute of Computer Science, Martin-Luther-University Halle-Wittenberg, Von-Seckendorff-Platz 1, 06120 Halle (Saale), Germany; 5 Core Facility Deep Sequencing, Martin-Luther-University Halle-Wittenberg, Magdeburger Str. 2, 06112 Halle (Saale), Germany; 6 German Centre for Integrative Biodiversity Research (iDiv) Halle-Jena-Leipzig, Deutscher Platz 5e, 04103 Leipzig, Germany; 7 Department of Community Ecology, Helmholtz Centre for Environmental Research – UFZ, Theodor-Lieser-Str. 4, 06120 Halle (Saale), Germany

**Keywords:** *Juncus effusus*, Soft rush, Helophyte, Wetlands, Transcriptome annotation, RNA-Seq, Polymorphism

## Abstract

**Background:**

*Juncus effusus* L*.* (family: Juncaceae; order: Poales) is a helophytic rush growing in temperate damp or wet terrestrial habitats and is of almost cosmopolitan distribution. The species has been studied intensively with respect to its interaction with co-occurring plants as well as microbes being involved in major biogeochemical cycles. *J. effusus* has biotechnological value as component of Constructed Wetlands where the plant has been employed in phytoremediation of contaminated water. Its genome has not been sequenced.

**Results:**

In this study we carried out functional annotation and polymorphism analysis of de novo assembled RNA-Seq data from 18 genotypes using 249 million paired-end Illumina HiSeq reads and 2.8 million 454 Titanium reads. The assembly comprised 158,591 contigs with a mean contig length of 780 bp. The assembly was annotated using the *dammit!* annotation pipeline, which queries the databases OrthoDB, Pfam-A, Rfam, and runs BUSCO (Benchmarking Single-Copy Ortholog genes). In total, 111,567 contigs (70.3%) were annotated with functional descriptions, assigned gene ontology terms, and conserved protein domains, which resulted in 30,932 non-redundant gene sequences. Results of BUSCO and KEGG pathway analyses were similar for *J. effusus* as for the well-studied members of the Poales, *Oryza sativa* and *Sorghum bicolor*. A total of 566,433 polymorphisms were identified in transcribed regions with an average frequency of 1 polymorphism in every 171 bases.

**Conclusions:**

The transcriptome assembly was of high quality and genome coverage was sufficient for global analyses. This annotated knowledge resource can be utilized for future gene expression analysis, genomic feature comparisons, genotyping, primer design, and functional genomics in *J. effusus*.

## Background

*Juncus effusus* L. (common, soft or mat rush) is an almost cosmopolitan monocotyledonous C3 plant that can grow abundantly in temperate wetlands, riparian strips, and other damp or wet terrestrial habitats [1]. The plant can vary substantially in morphological traits across its worldwide distributional range leading to the description of several subspecies. In Europe, only *J. effusus* ssp. *effusus* is known to occur but at least two genetically distinct cryptic lineages within the taxa have been found recently [2].

The plant grows in dense tufts and is able to reproduce by producing abundant seeds, which are easily dispersed, as well as via rhizomes, rendering the species an efficient colonizer [3]. The rhizomes as well as the shoots of this helophyte are characterized by forming aerenchyma for channeling air into the roots. This structural feature allows *J. effusus* to thrive in waterlogged environments [4–6]. The plant has multifarious effects on major element cycles in wetlands [7]. For example, radial oxygen loss can reduce CH_4_ production and increase CH_4_ oxidation in the rhizosphere [8–10]; on the other side, the input of organic carbon (root exudates and plant litter) can enhance methanogenesis [11, 12] and the aerenchyma can act as conduit for methane emission from organic-rich soils into the atmosphere [13].

Interactions of *J. effusus* with rhizospheric microbial communities as well as co-occurring plant species are exploited in ecotechnological applications such as Constructed Wetlands (CWs) [14]. CWs are means for wastewater treatment mirroring chemical transformation processes in natural wetlands to remove organic and inorganic contaminants from water [5]. Based on these characteristics *J. effusus* has been employed as a model plant in basic and applied research on wetland ecosystems [15–18]. The stem is of economic value as commodity for various woven products [19]. In addition, *J. effusus* has some medicinal properties and produces a variety of bioactive compounds [20, 21]. The pith of the stem, Junci Medulla, has been used in Chinese and other traditional medicines [22].

Understanding and quantifying genetic diversity within *J. effusus* is fundamental in predicting evolutionary pathways under changing environmental conditions. Marker systems such as single nucleotide polymorphisms (SNPs) and insertions/deletions (INDELs) have several advantages over conventional genetic markers. This includes their high genomic abundance, a co-dominant expression, and being mostly phenotypically neutral in nature [23]. Although a strong degree of genetic structuring has been suggested for *J. effusus* [2, 24], very little information is available at the molecular level. The species is diploid (2*n* = 42) and has a relatively small genome with a measured DNA 1C-value of 0.3 pg [25]. Based on this value the genome has an estimated size of approximately 270 Mbp, i.e. in between the genome sizes of *Arabidopis thaliana* (Arabidopsis Genome Initiative, 2000) and *Oryza sativa* [26, 27]. Plastome sequence data are available [28].

The aim of the present study was to develop a molecular database of *J. effusus* for enhanced research on natural and engineered wetland ecosystem functioning. To this end we employed RNA-Seq to record gene transcription in adult roots and shoots of 18 genotypes. The transcriptome was de novo assembled and annotated. Ortholog comparisons with phylogenetic relatives were carried out and the genetic diversity among the genotypes was evaluated based on a SNP analysis. The genomic information thus obtained will be of benefit for studies on wetland ecosystems and will foster further evolutionary studies on the Poales.

## Results

### Assembly of the *J. effusus* transcriptome

**Fig. 1:**
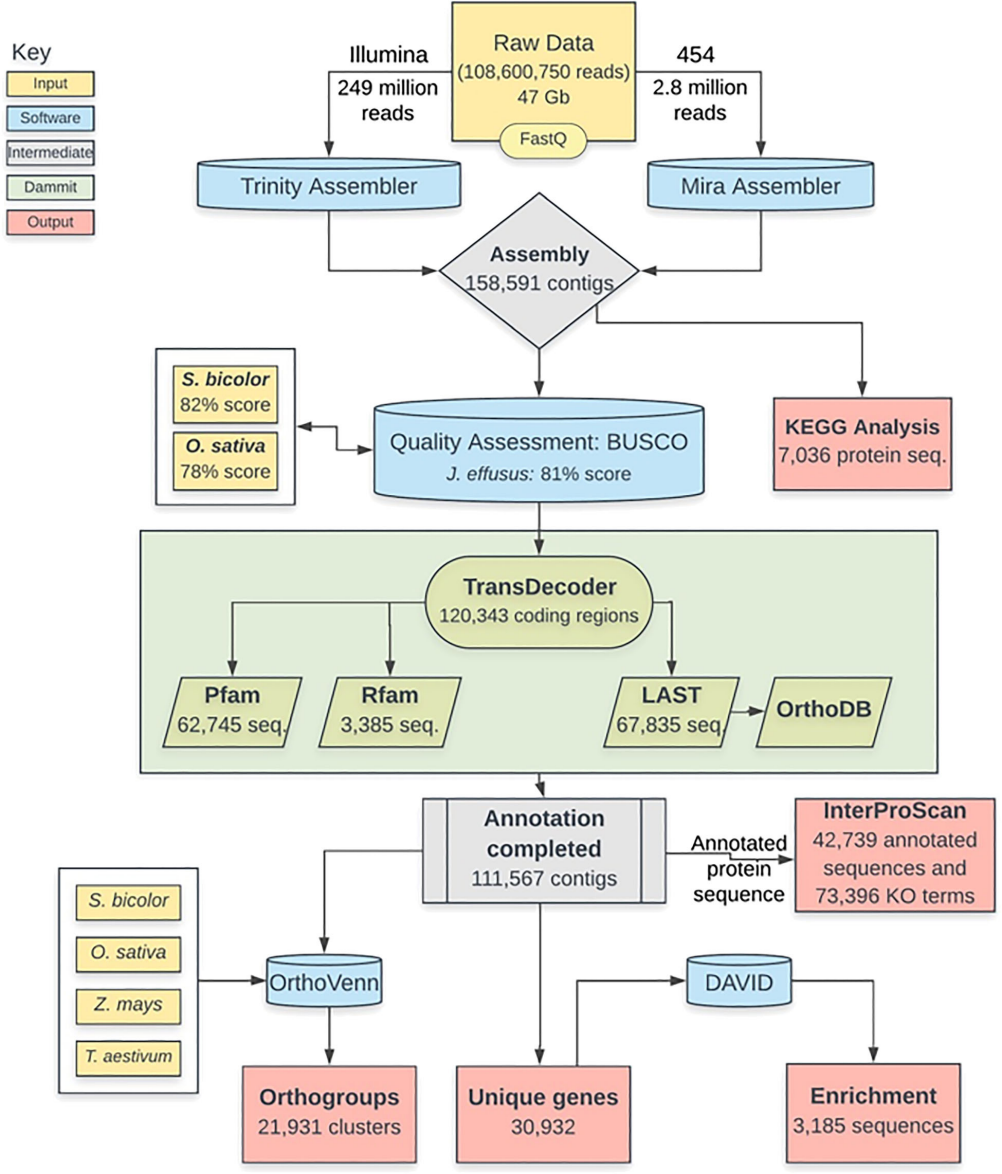
The overall process of transcriptome assembly, functional annotation, GO enrichment, orthologs clustering and validation

The overall process of transcriptome sequencing, assembly, annotation, ortholog clustering and validation of the assembly is summarized in Fig. 1. Illumina and 454 sequencing generated 108,600,750 clean reads comprising a total of 47 Gb, which was considered as good transcriptome coverage of the estimated genome size of around 270 Mb. The reads were de novo assembled using Trinity [29] and Mira [30, 31]. Quality analysis of the Trinity assembly with the software TransRate computed an optimized score of 0.34, which was better than the score for about 50% of 155 sampled de novo assembled transcriptomes [29]. CD-HIT [32, 33] was used to remove redundant sequences, which resulted in 158,591 contigs with lengths ranging between 200 bp to 18.5 kb. The average contig length was 780 bp, and N50 was 255 bp.

BUSCO v3 [34] was run on the *J. effusus* assembly as well as on previously assembled and annotated transcriptomes of *O. sativa* and *S. bicolor* to determine whether the genome coverage was sufficiently high to allow for comprehensive analyses. BUSCO results for the three species were very similar. Out of 429 single-copy ortholog genes common to the Eukaryota lineage there were 81, 82, and 78% complete single-copy BUSCOs, 42, 26, and 24% duplicated BUSCOs, 8.8, 4.1, and 6% fragmented BUSCOs, and 9.5, 12, and 15% missing BUSCOs respectively for *J. effusus*, *S. bicolor* and *O. sativa*.

### Constructing and annotating gene models

The assembled transcripts were annotated using Camille Scott’s *dammit!* annotation pipeline (https://github.com/camillescott/dammit). Gene model building using Transdecoder [35] predicted 120,343 likely coding regions (75.8% of all contigs) among which 79,203 (49.4%) contained a stop codon. There were 62,745 (39.6%) predicted coding regions that matched to the protein family database Pfam [36, 37], whereas a LAST search found that 67,835 predicted coding regions (42.8%) matched to the OrthoDB database [38, 39]. In addition, 3385 predicted coding regions (2.13%) matched to the Rfam database for non-coding RNAs [40]. In total, 111,567 contigs (70.3%) were annotated when combining results of all searches. The annotation features included putative nucleotide and protein matches, five- and three-prime UTRs, exons, mRNA, as well as start and stop codons.

**Fig. 2:**
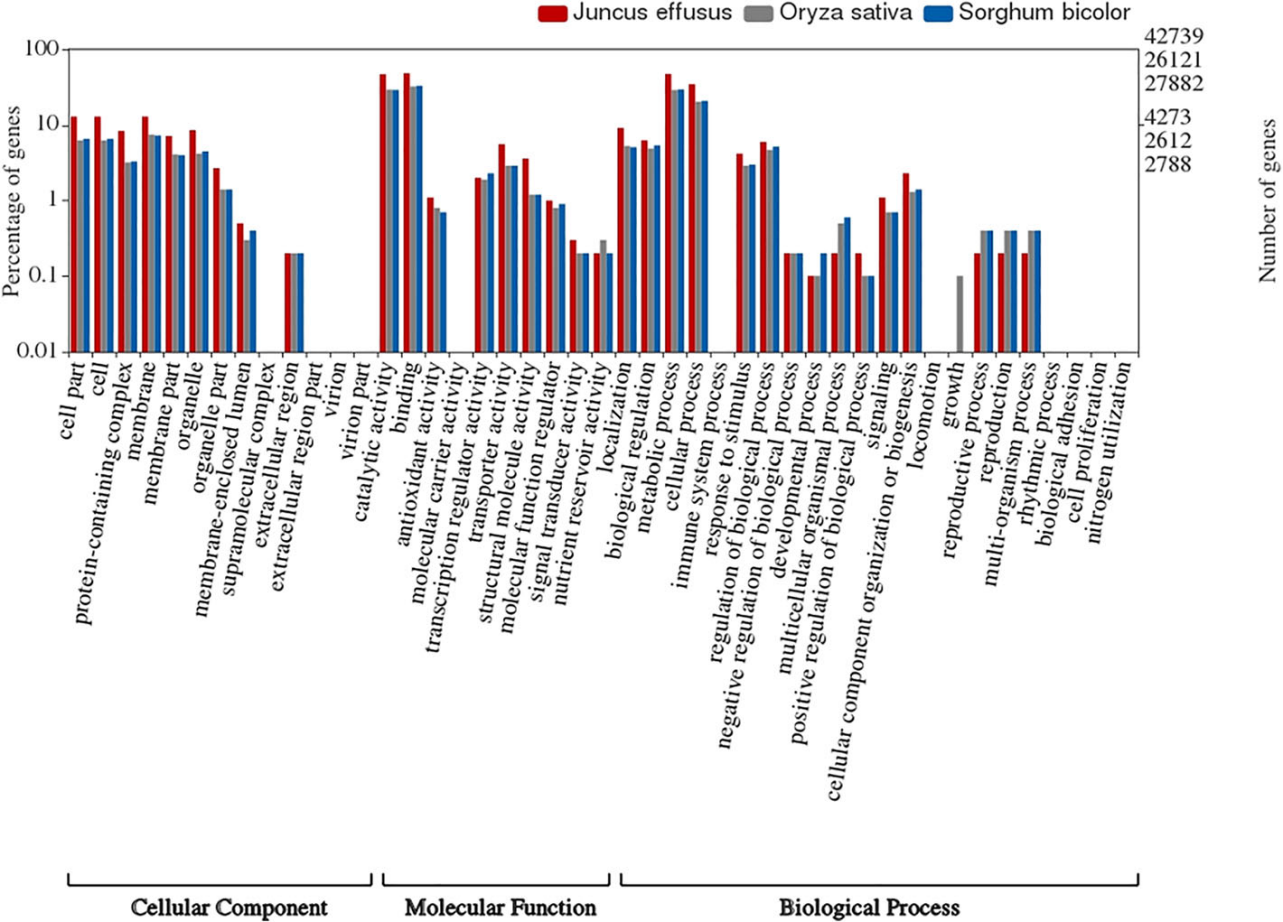
Histogram of level GO term assignments for *J. effusus*, *S. bicolor*, and *O. sativa* annotated gene models. Results are summarized for three main GO categories, *Cellular Component*, *Molecular Function*, *Biological Process*

To ensure further that the assembly was of high quality, we compared genomic features both statistically and manually with previously well-annotated transcriptomes of *S. bicolor* and *O. sativa*. GO analysis by InterProScan allowed classification of annotated transcripts into different functional groups. A total of 42,739 sequences (38.3% of all annotated contigs) were GO annotated out of the categories Molecular Functions, Cellular Components, and Biological Processes. The WEGO [41] plot for GO terms revealed that Molecular Functions was the dominant category (50.7% of all GO-annotations) followed by Biological Processes (35.7%) and Cellular Components (13.6%). Highly represented GO terms within Molecular Functions were ‘binding’ (GO:0005488) and ‘catalytic activity’ (GO:0003824); in the Biological Processes ontology group it were ‘cells’ (GO:0005623), ‘cellular process’ (GO:0009987), and ‘biological regulation’ (GO:0065007); and ‘cellular parts’ (GO:0044464) and ‘organelles’ (GO:0043226) in the Cellular Components ontology. The GO terms of the assembled transcriptome were compared with those of *S. bicolor* and *O. sativa* (Fig. 2). The results revealed a similar functional distribution with both reference transcriptomes, suggesting similar gene complements between *J. effusus* and its relatives. Minor contributions of ‘antioxidant activity’ (GO:0016209), ‘extracellular region’ (GO:0005576), ‘extracellular part’ (GO:0044421), and ‘viral reproduction’ (GO:0016032) were observed for *J. effusus*, while those categories were missing for *S. bicolor* and *O. sativa*.

KEGG analysis assigned enzyme commission (EC) numbers to 7036 protein sequences belonging to 380 different pathways. The KEGG category ‘metabolic pathways’ contained the majority of annotated proteins (851 members, 12.1%), followed by ‘biosynthesis of secondary metabolites’ (395 members, 5.61%). To evaluate further the qualitative accuracy of the functional annotation, we manually checked the completeness of the fundamental pathways photosynthesis, oxidative phosphorylation, glycolysis/gluconeogenesis, citrate cycle, pentose phosphate pathway, amino acid metabolism, and information processing. In addition, we checked the completeness of pathways involved in waterlogging [42]. All of those pathways were fully covered in the transcriptome.

**Fig. 3:**
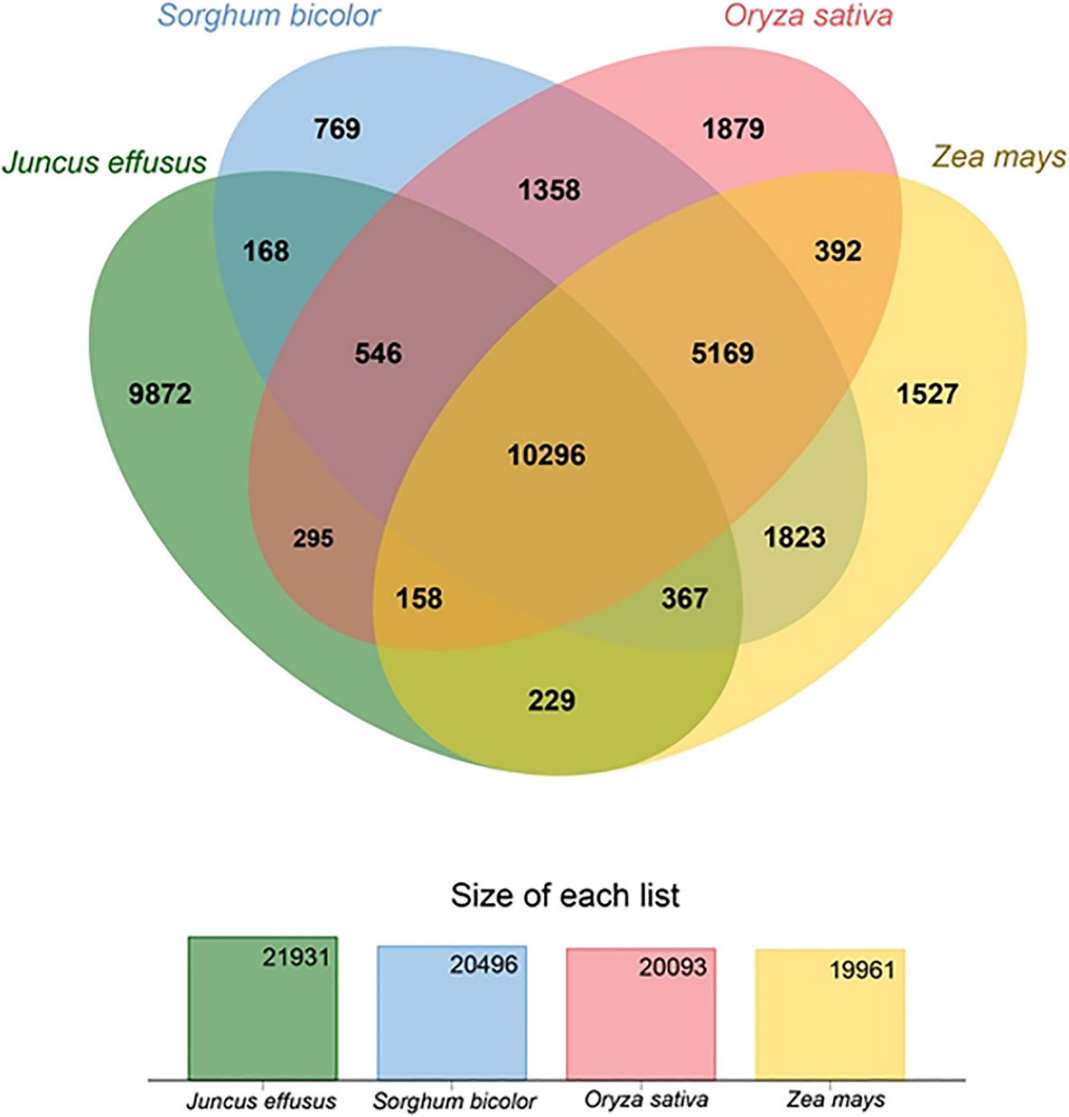
Comparisons of the core orthologous gene clusters among *J. effusus*, *O. sativa*, *Z. mays*, and *S. bicolor*

Clusters of orthologous gene (COG) analysis of *J. effusus* revealed the presence of 21,931 clusters, out of which 10,296 were shared among *S. bicolor*, *O. sativa*, and *Zea mays* (Fig. 3). These clusters involve proteins related to carboxylation and oxygenation, glycosylation, integral membrane components, nuclear mechanisms such as chromatin binding, cytoplasm and chloroplast integrities, and several other putative uncharacterized proteins. Further analyses of GO terms revealed a significant enrichment for the proteins related to electron carrier activities in the mitochondrial matrix (e.g., GO:0019243), photosystem II assembly (e.g., GO:0010207), transcription from plastid promoter (e.g., GO:0042793), regulation of protein dephosphorylation (e.g., GO:0035304), and hydrogen peroxide biosynthetic process (e.g., GO:0050665). The three members of the Poaceae had more similarities to each other than to *J. effusus*, which matches the topology of the phylogenetic tree based on plastome sequences [28]. Overall, 9872 clusters were unique for *J. effusus*, and included proteins involved in chloroplastic mechanisms, plasma membrane functioning, disease resistance, phytohormone productions for stress-ripening, and ion binding.

As a final quality control, we conducted Gene Set Enrichment Analysis (GSEA) with DAVID [43]. Results of GSEA were consistent with the KEGG findings. A complete list of enriched sequences and number of KEGG orthology (KO) hits for *J. effusus*, *S. bicolor*, *O. sativa* and *Z. mays* is presented in Table 1. Sequences of *J. effusus* with redundant KO terms likely originating from paralogous genes and orthologues in the various genotypes were combined to a total of 30,932 gene sequences with matching hits to proteins (E < 1e^− 6^). Among these sequences there were 3185 enriched sequences (10.2%) of which most belonged to the sub-groups of metabolic pathways (1407 sequences, 44.1%), biosynthesis of secondary metabolites (1140 sequences, 35.8%), biosynthesis of amino acids (156 sequences, 4.89%), oxidative phosphorylation (114 sequences, 3.57%), amino sugar and nucleotide sugar metabolism (113 sequences, 3.54%). Sequences grouping into the category genetic information processing accounted for 322 sequences (1.04%) and included the enriched categories ribosomes (276 sequences, 85.7%) and protein export (46 sequences, 14.2%). By contrast, environmental information processing (EIP) contained no enriched KEGG pathways for *J. effusus* (although the EIP pathways were complete as mentioned above). All pathways enriched for in *J. effusus* were also enriched for in *S. bicolor* except porphyrin and chlorophyll metabolism, which was only enriched in *J. effusus*.

**Table 1:** Genes enriched for KEGG and number of KO hits for *Juncus effusus*, *Sorghum bicolor*, *Oryza sativa*, and *Zea mays*

		*J. effusus*		*S. bicolor*		*O. sativa*		*Z. mays*	
Ranking	KEGG pathway	KEGG hits	KO hits	KEGG hits	KO hits	KEGG hits	KO hits	KEGG hits	KO hits
1.	Metabolism								
1.0	Global and overview maps								
1100	Metabolic pathways	1407	855>	1431	865	1369	817	1793	850
1110	Biosynthesis of secondary metabolites	833	396	844	395	776	397	1033	400
1130	Biosynthesis of antibiotics	307	193	312	194	352	192	462	194
1200	Carbon metabolism	–	–	–	–	226	90	263	90
1230	Biosynthesis of amino acids	156	98	157	98	189	97	–	–
1.1	Carbohydrate metabolism								
00010	Glycolysis / Gluconeogenesis	92	33	94	33	112	32	134	33
00020	Citrate cycle (TCA cycle)	–	–	–	–	49	20	–	–
00030	Pentose phosphate pathway	–	–	–	–	46	17	–	–
00053	Ascorbate and aldarate metabolism	–	–	–	–	37	16	–	–
00500	Starch and sucrose metabolism	–	–	–	–	107	30	–	–
00520	Amino sugar and nucleotide sugar metabolism	113	40	114	40	105	40	–	–
00620	Pyruvate metabolism	–	–	–	–	73	27	–	–
1.2	Energy metabolism								
00190	Oxidative phosphorylation	114	86	118	91	–	–	129	86
00195	Photosynthesis	–	–	–	–	75	35	–	–
00710	Carbon fixation in photosynthetic organisms	–	–	–	–	70	25	–	–
1.3	Lipid metabolism							–	–
00073	Cutin, suberine and wax biosynthesis	–	–	–	–	–	–	28	8
00100	Steroid biosynthesis	–	–	–	–	–	–	38	18
00591	Linoleic acid metabolism	–	–	–	–	–	–	15	4
1.5	Amino acid metabolism								
00260	Glycine, serine and threonine metabolism	52	34	52	34	–	–	–	–
00330	Arginine and proline metabolism	–	–	–	–	–	–	62	24
00350	Tyrosine metabolism	–	–	–	–	–	–	40	18
1.6	Metabolism of other amino acids								
00480	Glutathione metabolism	98	18	98	18	–	–	–	–
1.7	Glycan biosynthesis and metabolism								
00510	N-Glycan biosynthesis	–	–	–	–	–	–	44	31
1.8	Metabolism of cofactors and vitamins								
00730	Thiamine metabolism	13	11	14	11	–	–	–	–
00770	Pantothenate and CoA biosynthesis	–	–	–	–	–	–	30	16
00860	Porphyrin and chlorophyll metabolism			41	33	36	33	50	33
1.9	Metabolism of terpenoids and polyketides								
00900	Terpenoid backbone biosynthesis	–	–	–	–	–	–	58	30
00904	Diterpenoid biosynthesis	–	–	–	–	27	18	–	–
1.10	Biosynthesis of other secondary metabolites								
00940	Phenylpropanoid biosynthesis	–	–	–	–	114	17	–	–
2.	Genetic Information Processing								
2.1	Transcription								
03040	Spliceosome	–	–	–	–	–	–	194	102
2.2	Translation								
03010	Ribosome	276	130	278	132	–	–	–	–
03015	mRNA surveillance pathway	–	–	–	–	97	49		
2.3	Folding, sorting and degradation								
03060	Protein export	46	26	47	26	–	–	–	–
04122	Sulfur relay system	–	–	–	–	–	–	14	10
3.	Environmental Information Processing								
3.2	Signal transduction								
04075	Plant hormone signal transduction	–	–	–	–	172	38	253	41
4.	Cellular Processes								
4.1	Transport and catabolism								
04144	Endocytosis	–	–	–	–	117	58	–	–

### Detection of nucleotide polymorphisms

We identified 566,433 polymorphisms (478,627 SNPs and 87,806 INDELs) in 99,692 contigs. On average over the whole transcriptome, one polymorphism was identified in 174 bp. This relative abundance is similar to SNPs frequency in *O. sativa* (1 per 147 bp) [44] and *Z. mays* (1 per 200 bp) [45]. Among the contigs having at least one polymorphism, 35,429 were assigned to KO terms and comprised 3521 unique KO identifiers. The polymorphism distribution among gene functions was non-random. Ten out of the top 20 KO IDs with the highest polymorphism frequencies (ranging from 9 to 49 per KO ID) were associated with plant defense mechanisms and wound healing (disease resistance protein RPM1, peroxidase, chalcone synthase, ATP-binding cassette of multidrug resistant transporter, phenylalanine ammonia-lyase, laccase, glutathione *S*-transferase, *trans*-resveratrol di-*O*-methyltransferase, cinnamyl-alcohol dehydrogenase, ionotropic glutamate receptor). Most of the remaining KO IDs with ≥5 polymorphisms (50 IDs in total) were involved in cytoskeleton formation, repair mechanisms associated with replication, transcription and translation, as well as metabolism of the small amino acids Gly, Met, Cys, Ser and Thr.

In order to experimentally confirm the suitable of the SNP dataset for genotyping of *J. effusus*, we selected 44 SNPs for loci amplification via PCR and subsequent Sanger sequencing. The loci chosen were predicted to be associated with nitrogen assimilation (Table 2), which is an important function in wastewater treatment via constructed wetlands. Out of those selected a total of 35 (80%) loci could be successfully PCR-amplified and thus the respective SNPs confirmed by sequencing.

**Table 2:**
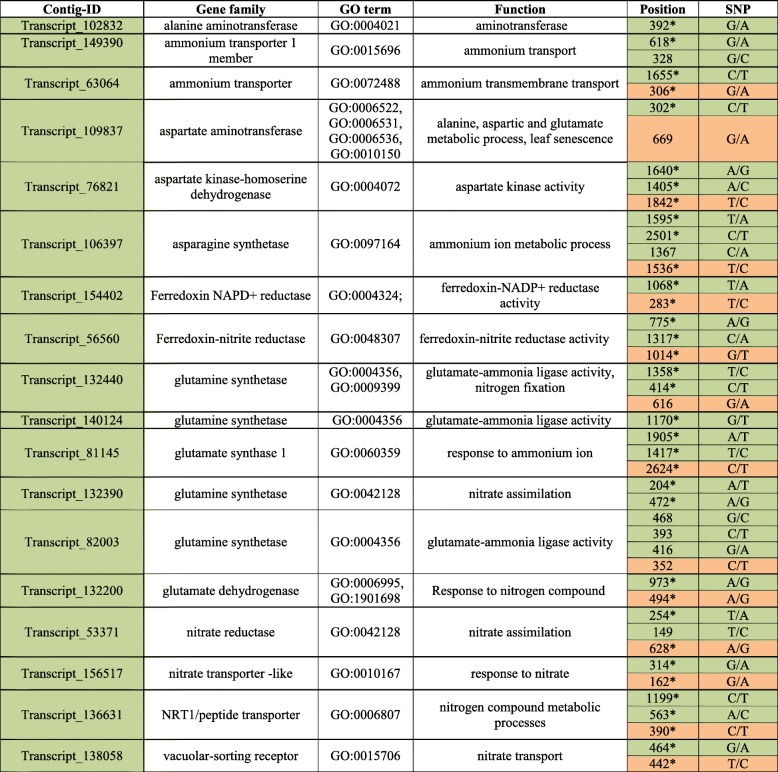
Candidate genes putatively linked to N assimilation and associated SNP loci

SNPs marked in orange are located at synonymous codon-positions. An asterisk marks successful PCR amplification

## Discussion

In the past the extensive research on and the manifold biotechnological applications of the common wetland plant *J. effusus* were carried out without *omics*-based knowledge. Information on nucleic acid sequences was restricted to the plastome [28] and several chromosomal microsatellite loci, the latter of which formed the basis for recent studies on the intraspecific variability of genotypes [46–48]. In order to develop a more comprehensive inventory of genetic information of *J. effusus* we opted for transcriptome profiling of 18 genotypes via RNA-Seq. The geographical origins of the genotypes cover a substantial portion of the European distribution range of this species. RNA-Seq has been used for other members of the Poales, and particular of members of the family Poaceae, for various purposes such as de novo sequencing and assembly (rice, [49]), querying the transcriptome profiles of distinct tissues and at various development stages (wheat; [50]), characterization of genes involved in specific biochemical pathways (cordgrass, [51]; pineapple, [52]), identification of novel transcriptome sequences (maize, [53]) and isoforms (false-brome, [54]), SNP analysis (wheat, [55]), and simple sequence repeats detection (bamboo, [56]).

In this study we carried out both single and paired-end sequencing runs to improve the de novo assembly [57]. The assembly was deemed successful based on a good TransRate score of 0.34 as well BUSCO results that were quite similar to those of the closer relatives *S. bicolor*, *O. sativa*, and *Z. mays*. Annotation was performed using the *dammit!* pipeline (prepared by Camille Scott https://github.com/dib-lab/dammit), which is one of two pipelines available for transcriptome annotation known to us. The other pipeline, annocript [58], was in its earlier stage of development at the time of annotating the *J. effusus* transcriptome and it did not include information on lower hierarchy of GO terms. The *dammit!* pipeline includes quality assurance step via BUSCO, executes gene model building [59], and compares each transcript against entries in several databases [e.g. protein domains [60], non-coding RNAs [40], ortholog matches and orthology assignments [61]].

In total, 70% of the contigs (111,567) were annotated for functional descriptions, GO terms, and conserved protein domains. Sequence similarity searches and gene model building revealed the presence of 120,343 likely coding regions, which were computationally condensed to non-redundant 30,932 gene sequences with matching hits to proteins (E < 1e^− 6^). The GO annotation of *J. effusus* transcriptome sequences revealed a similar functional distribution with the reference transcriptomes of *S. bicolor* and *O. sativa*, which evinces overlapping gene complements between *J. effusus* and its relatives. As expected from transcriptome analyses of other members of the Poacaea the most highly represented GO terms belonged to the Molecular Functions category. KEGG pathway analyses mirrored these similarities, and COG analysis matched the topology of the phylogenetic tree based on plastome sequences. Overall, gene function analyses showed that predicted protein sequences exhibited high coverage of KEGG pathways (i.e. 380 KEGG pathways were identified with 7036 enzyme codes). The sequences that had no significant matches may be lacking a known conserved functional domain or they were too short to have a significant sequence match. These sequences might be of potential interests for future research on novel gene products, alternative splice variants, and differentially expressed genes.

A further aim of this study was to identify nucleotide polymorphism markers that can be readily used for genotyping of *J. effusus*. SNPs frequency in the transcriptome (1 in 147 bp) was similar to those of other members of the Poales. SNPs may not be distributed evenly across the genome positionally and functionally. For example, in soy bean SNPs were found to occur more frequently at the chromosome ends putatively as a consequence of diversifying recombination and mutation events [62]. Without reference genome for *J. effusus*, however, the chromosomal distribution of the found SNPs cannot be assessed properly. At the functional level, we found the highest SNP frequency per locus in transcripts involved in plant defense mechanisms and wound healing. In *O. sativa*, a large amount of SNP variation was found in genes involved in in stress response and other processes associated with adaptation to a changing environment [63]. Similarly, for *A. thaliana* it has been suggested that the maintenance of variation in defense mechanisms may be associated with the species’ persistence in environmentally heterogeneous habitats [64]. Hence, also *J. effusus* as a wetland indicator plant and tolerating a wide range of ecological conditions may benefit from a diverse genomic background associated with stress tolerance. The polymorphisms identified here at transcriptome level will help explain adaptive mechanisms shaping and maintaining the complex intra-specific differentiation pattern described for this species [24].

## Conclusions

This study is the first genomics analysis of *J. effusus*. The assembly and annotation were considered as of high quality. The results are expected to open new opportunities for future *omics* studies and population genetics of this common wetland plant.

## Materials and methods

### Plant materials and RNA isolation

**Fig. 4:**
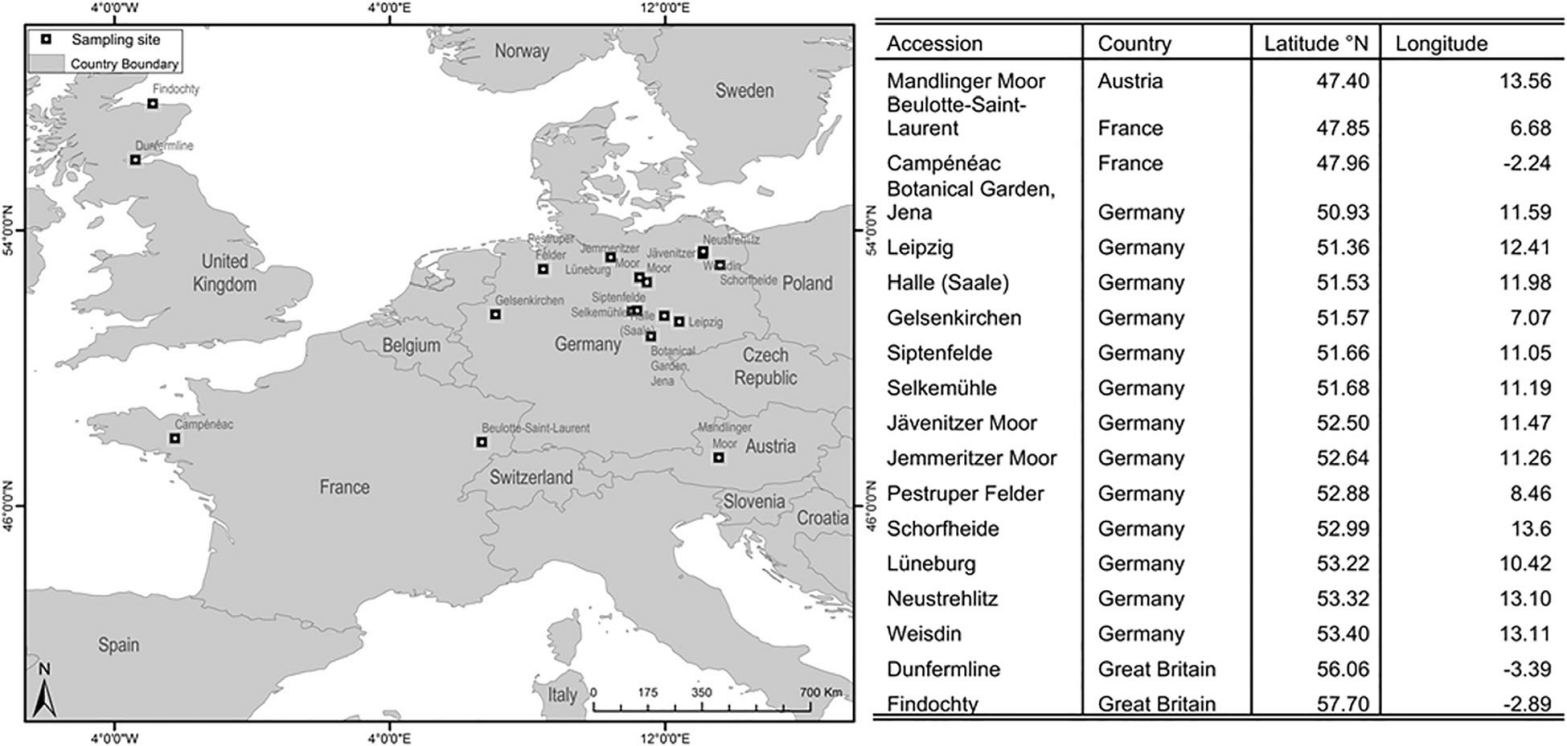
Overview map indicating sampling sites for *J. effusus* ecotypes analysed in this study

Plant tissues (roots and shoots) were harvested from individuals at the vegetative developmental stage that were raised from seeds collected in the field. In total 18 genotypes were used. The geographical distribution of the sampled locations is presented in Fig. 4. The obtained plant tissues were snap-frozen in liquid nitrogen and kept at − 80 °C until processing. Total RNA was extracted from roots and shoots separately using the RNeasy Plant Mini Kit according to the manufacturer’s protocol (Qiagen, Hilden, Germany). In order to represent a wide range of expressed genotypic variability within individuals and the species, extracts were then pooled with the final mix containing approximate equal contributions of each genotype and tissue type.

### Transcriptome sequencing and assembly

Standard library preparation and sequencing of total RNA using one lane of an Illumina HiSeq (2 × 100 bp PE) and two runs of Roche 454 Titanium was done at the Duke Center for Genomic and Computational Biology (Durham, USA) yielding 249 million Illumina PE reads and 2.8 million 454 reads. After removing sequencing adaptors, quality-controlled reads were processed using two different de novo transcriptome assemblers. Illumina reads were assembled using Trinity version 20,130,225 [29], and 454 reads were assembled using Mira version 3.9.15, [30, 31]. Both assemblers were run with default parameters. The software TransRate [65], which enables reference-free quality evaluations of de novo transcriptome assemblies, was used for analysis of Trinity assembly. Mira and Trinity assemblies were combined and CD-HIT version 4.5.7 [32, 33] was used to remove redundant sequences.

### Functional annotation

We used Camille Scott’s *dammit!* annotation pipeline to annotate the transcriptome assembly (https://github.com/camillescott/dammit). Within the pipeline, annotation begins by building gene models with TransDecoder v2.0.1 [35]. Subsequently, it utilizes multiple databases for annotating the transcriptome: protein domains in Pfam-A v29.0 [36, 37], Rfam v12.0 to find non-coding RNAs [40], the execution of a LAST search for known proteins in the OrthoDB database [61], ortholog matches in the BUSCO database [34], and orthology searches in OrthoDB [61]. The assembly quality and annotation completeness were assessed using BUSCO v3, which supports interpretation of assembly coverage based on the presence of single-copy orthologous genes [34]. To compare the assembly results of *J. effusus*, BUSCO was also run with transcriptomes of *O. sativa* subsp. *japonica* and *S. bicolor* (http://plants.ensembl.org/info/website/ftp/index.html).

### Functional classification

Gene ontology (GO) analyses were carried out on predicted protein sequences using InterProScan v.5.26–65.0, available as virtual machine image on Jetstream cloud (https://use.jetstream-cloud.org/application/images/586). The GO annotations were then plotted using the BGI-WEGO program (http://wego.genomics.org.cn/) together with *O. sativa* and *S. bicolor* to elucidate relative distribution of Molecular Function, Cellular Components, and Biological Processes [41]. Afterwards, predicted protein sequences were mapped to the reference canonical Kyoto Encyclopedia Genes and Genomes (KEGG) pathways as additional approach for functional annotation and categorization. The predicted protein sequences were submitted to the KEGG automatic annotation server (KAAS) (http://www.genome.jp/tools/kaas/) with the single-directional best-hit (SBH) method selected for pathway mapping. Subsequently, gene set enrichment analysis (GSEA) was performed on non-redundant gene sequences using the GO based enrichment tool DAVID (Database for Annotation, Visualization and Integrated Discovery) [43]. DAVID provides ranking of KEGG pathways on the basis of Benjamini corrected *p*-values. The number of genes shared between *J. effusus* and the members of the Poaceae *S. bicolor*, *O. sativa*, and *Z. mays* were assessed by OrthoVenn, a web platform that identifies COGs clusters by comparing the predicted proteins sequences with the database [66]. Default parameters were used for protein similarity comparisons.

### Polymorphism identification

Paired end Illumina reads were mapped using segemehl version 0.1.9 (Hoffmann et al., 2009) against the de novo transcriptome assembly allowing two mismatches as well as multiple mappings to redundant sequences in the variable mapping seed. Subsequently single nucleotide polymorphisms (SNPs) and insertions/deletions (INDELS) were detected using samtools mpileup (version 1.1) [67] using default parameters. SNPs were annotated using Ensemble Variant Effect Predictor (version 75) [68]. Contigs with significant SNPs (3-fold greater abundance than the transcriptome-wide average) were identified and then KO assigned using *concatenate* and *merge* function in R-statistical language. Lastly, KO terms were annotated by using the online KEGG database (https://www.genome.jp/kegg/ko.html). Selected SNP loci putatively associated with N assimilation were verified by the following approach: For the same 18 genotypes used for total RNA extraction, genomic DNA was extracted using the DNeasy Plant Mini Kit according to the manufacturer’s protocol (Qiagen, Hilden, Germany). Primers for each locus were designed using a Primer3 online implementation (http://bioinfo.ut.ee/primer3-0.4.0/) targeting a size range between 300 and 600 bp. Amplification of loci was conducted in a total volume of 20 μL including 5 pMol of each locus-specific forward and reverse primer, 200 μM dNTP, 2 *μ*L 10x DreamTaq-buffer (Thermo Fischer Scientific), 0.8 U DreamTaq-Polymerase and approx. 5 ng DNA. The PCR program involved 3 min at 95 °C, then 40 cycles of 95 °C for 30 s, 58–62 °C for 40 s, 72 °C for 1 min and a final 10 min of 72 °C. PCRs products were purified by centrifuging for 3 min at 2800 rpm through cross linked dextran gel (Sephadex G-50 Superfine, GE Healthcare Life Sciences, Germany). PCR-products were directly cycle-sequenced from both ends using the ABI BigDye Terminater v3.1 cycle sequencing Kit using the same primers. Products were sequenced on an Applied Biosystems 3130xl Genetic Analyzer (Applied Biosystems, Foster City, USA).

## Data Availability

The assembly is available at GenBank under accession no. PRJNA345287. All annotation files incl. Supplementary Tables are available at Mendely under the DOI:10.17632/cx7k2v38m7.3.

## Abbreviations

BUSCO: Benchmarking Single-Copy Ortholog genes
EIP: environmental information processing
GSEA: Gene Set Enrichment Analysis
INDELs: insertions/deletions
SNPs: single nucleotide polymorphisms
UTR: untranslated region
